# High Infestation by *Dawestrema cycloancistrioides* in *Arapaima gigas* Cultured in the Amazon Region, Peru

**DOI:** 10.1155/2014/245878

**Published:** 2014-11-30

**Authors:** Patrick D. Mathews, Antonio F. Malheiros, Narda D. Vasquez, Milton D. Chavez

**Affiliations:** ^1^Department of Parasitology, Institute of Animal Biology, University of Campinas, 13083-862 Campinas, Brazil; ^2^Department of Biological Science, University of State of Mato Grosso, 78200-000 Cáceres, Brazil; ^3^Department of Tropical Aquaculture, Institute of Biology, National University of the Peruvian Amazon, 765 Iquitos, Peru; ^4^Department of Health, Safety and Environment, Enersul Limited Partnership, Calgary, Canada T2H 1M5

## Abstract

The aim of this study was to evaluate the presence of *Dawestrema cycloancistrioides* in semi-intensive fish farming of fingerlings of *Arapaima gigas*. Between September and November 2013, 60 individuals of *A. gigas* born in captivity, were collected in three concrete ponds, from a semi-intensive fish farm in the Peruvian Amazon. For the study of sclerotized structures, parasites were fixed in a solution of ammonium picrate glycerine and mounted in Canada balsam. To visualize internal structures, parasites were fixed in hot formaldehyde solution (4%) for staining with Gomori's trichrome. The parasitic indexes calculated were prevalence, mean intensity, and mean abundance. This study identified a high infestation of a monogenean *D. cycloancistrioides* in gills of *A. gigas*. The prevalence was 100%. The mean intensity and mean abundance of the parasite were 144.9 of parasites per individual. This study confirms the necessity of constant monitoring of fish in order to reduce fish mortality.

## 1. Introduction

The* Arapaima gigas* is an endemic species of the Basin Amazon and is considered one of the largest freshwater fish in the world. The* A. gigas* can reach up to three meters in length and 200 kg of total weight [1] and is a much appreciated species with great acceptance in the Amazonian market being regarded as a protein source of the highest quality. However, due to its high nutritional demand for the population of the Amazon region, in recent years the natural stocks of this fish have suffered drastic reduction [2]. Farming paiche is thus a possible solution to the overexploitation of this species in many rivers of the Amazon. However, to allow the breeding, it turns out that the necessity to solve the problems regarding diseases and parasites upsurge, which are affecting this species in controlled environments as a consequence of intensive farming under inadequate management.

The dactylogyrid monogeneans are ectoparasites usually attached to the gills of fish. Like other monogeneans, dactylogyrids have a direct life cycle and can easily multiply and disperse under fish culture conditions, reaching very high intensities. These monogeneans have been linked to major losses in fish culture [3, 4]. Recently, studies in several species of farmed fishes in the Amazon region of Peru showed a high infestation by monogeneans species, indicated as the probable cause of high mortality [5–8].

Therefore, with the gradual increase of intensive and semi-intensive fish farming in the Peruvian Amazon, there is a need for constant monitoring of the fish for the diagnosis and timely control of infestations by monogeneans. In this sense, the present study aims to evaluate the monogenean infestation in* A. gigas* bred in a fish farm in the Peruvian Amazon.

## 2. Materials and Methods

### 2.1. Study Site and Animals

**Figure 1:**
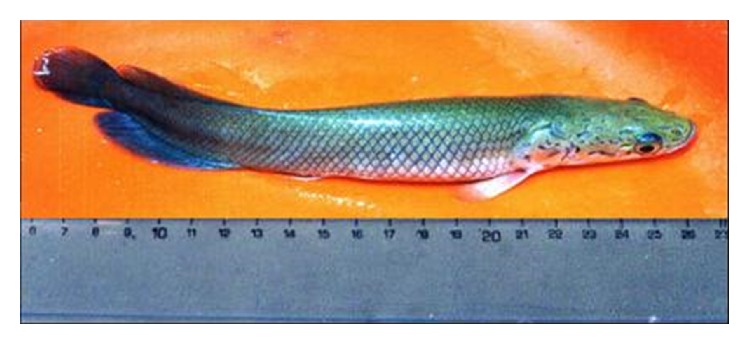
Specimen of* Arapaima gigas* collected from cultured ponds.

Between September and November 2013, the period which corresponds to the relative dry season, 60 individuals of the species* A. gigas* (20.40 ± 0.10 cm of length and weight of 76.06 ± 0.86 g), born in captivity (Figure 1), were collected in three concrete ponds, from a semi-intensive fish farm, belonging to the fish culture station Quistococha Research Center, located between the cities Iquitos and Nauta, northeast of Department of Loreto, Peru (3°48′48.9′′ N and 073°19′18.2′′ W). The relative humidity in this region varies between 80% and 100%. Annual rainfall ranged between 1500 and 3000 mm at 328 mean sea level.

### 2.2. Physicochemical Parameters of the Water

The parameters were measured three times daily (at 7 AM and noon and at 5 PM) with daily checks of dissolved oxygen, pH, temperature, and conductivity by means of an YSI multiparameter meter (Model MPS 556). Ammonium values, hardness, carbon dioxide, and total alkalinity were monitored weekly in the morning at 8 AM, using a complete package for analysis of freshwater (LaMotte AQ-2).

### 2.3. Parasitological Analysis

Following that, the fish were sacrificed by cerebral puncture and placed in individual containers. For examination of the gills, the samples were separated and placed in glass containers with a 1 : 4.000 formalin solution. After one hour, the gills were stirred in the same solution and then removed from the containers. Helminthes were allowed to settle on the bottom of the glass containers in the remaining formalin solution and subsequently collected with the aid of a small probe and a dissecting microscope (Nikon SM-30). For the study of sclerotized structures, parasites were fixed in a solution of ammonium picrate glycerine (GAP) and mounted in Canada balsam according to Thatcher [9]. Some specimens were mounted unstained in Gray and Wess' medium. To visualize internal structures, parasites were fixed in hot formaldehyde solution (4%) for staining with Gomori's trichrome. The identification of the parasites was based on the methodology of Thatcher [9] and Kritsky et al. [10].

The parasitic indexes calculated for assessing the level of infestation of parasites in the fish, prevalence (number of hosts infected divided by the total number of hosts in a sample), mean intensity (total number of parasites divided by number of hosts infected), and mean abundance (number of parasites divided by the total number of hosts in a sample) are the same as those described in Bush et al. [11]. The research was authorized by the Research Institute of the Peruvian Amazon.

## 3. Results

**Figure 2:**
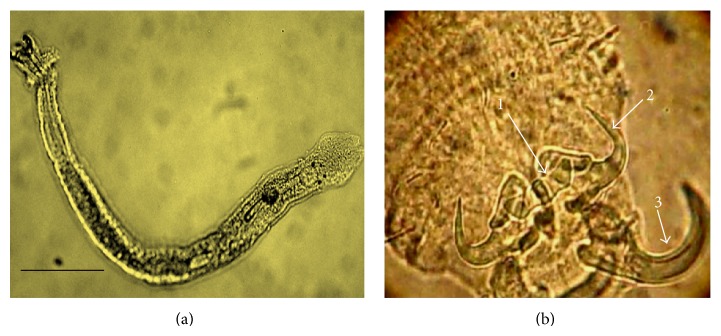
*Dawestrema cycloancistrioides *(a) total. (b) Posterior region, 1—ventral bar, 2—ventral anchor, 3—dorsal anchor. Scale bars = 10 *μ*m.

The moribund fishes collected for analysis were in emaciated condition. Due to the accumulation of mucus, the gills were pale and viscous. The necropsy of fingerlings from* A. gigas* evidenced the infestation by* Dawestrema cycloancistrioides* in the gill filaments of the fish (Figure 2). Indeed, the totality of the examined fish (60) showed a high parasitic infestation (Table 1).

In the present study, the values of the physicochemical parameters of the water from the culture ponds were dissolved oxygen (5.74 ± 0.8 mg L^−1^), pH (6.84 ± 0.10), temperature (24 to 30°C) and conductivity (106.1 ± 14.0 *μ*s cm^−1^), ammonium values (0.20 ± 0.10 mg L^−1^), hardness (20.40 ± 1.60 mg L^−1^), carbon dioxide (3.1 ± 0.6 mg L^−1^), and total alkalinity (16.12 ± 0.80 mg L^−1^).

## 4. Discussion

In intensive fish farming, diseases outbreaks are a major concern to the farmers because in such systems fish are exposed to a high number of stressors (poor water quality, crowding, manipulation, breeding, and transport) which may negatively affect their immune system and disease resistance [12, 13]. Water temperature of the fish ponds presented a strong variation during the present study, reaching its lowest values (24°C) during the first hours of the day and the highest values (30°C) between noon and three hours later. In the same sense, in our study the concentration of ammonia, found in the ponds of cultivated* A. gigas*, was not within the expected range of values for tropical species. In this context, the fish are subjected to stress thus becoming more susceptible to infestation by parasites and reduced disease resistance [14, 15]. We suspect that the high parasitism by monogeneans was due to the imbalance in the homeostasis of fish.

Parasites that have a direct life cycle, such as monogeneans, are more frequently found in lentic environments and this type of environment favors the transmission of these parasites [3]. In regions with tropical and semitropical climates, the life cycle of monogenean can be completed in less than one day and the monogeneans proliferate rapidly [3, 9]. The climate in the region of this study is tropical humid with annual average temperature of 28.3°C and relative humidity of 85%, favoring the speed of life cycle. In the earthen ponds where fish were collected, the water circulation is almost negligible or nonexistent and the same have a high density of fish population. These drawbacks favor the contact with the monogeneans [3] and may justify the fact that the fishes have shown elevated prevalence of monogenean.

In* A. gigas* three species of monogeneans assigned to* Dawestrema* genus (*D*.* cycloancistrium*,* D*.* cycloancistrioides, *and* D*.* punctatum*) have been reported from natural environments and two in specimens from fish farming (*D. cycloancistrium* and* D. cycloancistrioides*) [9, 10, 16, 17], evidencing a high specificity of* Dawestrema* species parasitizing* A. gigas*. However, this specificity may be related to the fact that many of monogeneans which parasitize fish are host-specific, because they have co-evolved with their hosts [18].

In the present study,* D. cycloancistrioides* showed high parasitism along with prevalence rates of 100%. Our results agree with Hargreaves and Tucker [15], who reported similar prevalence, with mean intensity and mean abundance of 280 for specimens of* A. gigas* collected from fish farming in Brazil. In addition, Gross et al. [14] reported a prevalence of 100% from an aquarium in the United States, confirming the occurrence of this parasite in cultivated* A. gigas*.

## 5. Conclusion

The results of this study and studies addressing various aspects of parasites in other cultivated species confirm the necessity of constant monitoring of fish, seeking the diagnosis and timely control of infestations by monogeneans, in order to reduce fish mortality.

## Figures and Tables

**Table 1:** Parasitic indexes of *Dawestrema cycloancistrioides* in juveniles of paiche (*Arapaima gigas*) cultured in the Peruvian Amazon.

Parasitic indexes	*Dawestrema cycloancistrioides *
Prevalence (%)	100
Abundance	8695
Mean abundance	144.9
Mean intensity	144.9
